# The endotoxin hypothesis of neurodegeneration

**DOI:** 10.1186/s12974-019-1564-7

**Published:** 2019-09-13

**Authors:** Guy C. Brown

**Affiliations:** Department of Biochemistry, University of Cambridge, Cambridge, CB2 1QW UK

**Keywords:** Endotoxin, Neurodegeneration, Alzheimer’s disease, Parkinson’s disease, Microglia, Inflammation, Neuroinflammation, Lipopolysaccharide, Gut microbiome, Bacteria

## Abstract

The endotoxin hypothesis of neurodegeneration is the hypothesis that endotoxin causes or contributes to neurodegeneration. Endotoxin is a lipopolysaccharide (LPS), constituting much of the outer membrane of gram-negative bacteria, present at high concentrations in gut, gums and skin and in other tissue during bacterial infection. Blood plasma levels of endotoxin are normally low, but are elevated during infections, gut inflammation, gum disease and neurodegenerative disease. Adding endotoxin at such levels to blood of healthy humans induces systemic inflammation and brain microglial activation. Adding high levels of endotoxin to the blood or body of rodents induces microglial activation, priming and/or tolerance, memory deficits and loss of brain synapses and neurons. Endotoxin promotes amyloid β and tau aggregation and neuropathology, suggesting the possibility that endotoxin synergises with different aggregable proteins to give different neurodegenerative diseases. Blood and brain endotoxin levels are elevated in Alzheimer’s disease, which is accelerated by systemic infections, including gum disease. Endotoxin binds directly to APOE, and the APOE4 variant both sensitises to endotoxin and predisposes to Alzheimer’s disease. Intestinal permeability increases early in Parkinson’s disease, and injection of endotoxin into mice induces α-synuclein production and aggregation, as well as loss of dopaminergic neurons in the substantia nigra. The gut microbiome changes in Parkinson’s disease, and changing the endotoxin-producing bacterial species can affect the disease in patients and mouse models. Blood endotoxin is elevated in amyotrophic lateral sclerosis, and endotoxin promotes TDP-43 aggregation and neuropathology. Peripheral diseases that elevate blood endotoxin, such as sepsis, AIDS and liver failure, also result in neurodegeneration. Endotoxin directly and indirectly activates microglia that damage neurons via nitric oxide, oxidants and cytokines, and by phagocytosis of synapses and neurons. The endotoxin hypothesis is unproven, but if correct, then neurodegeneration may be reduced by decreasing endotoxin levels or endotoxin-induced neuroinflammation.

## Background

Neurodegeneration is progressive damage and death of neurons, normally as a result of neurodegenerative diseases, such as Alzheimer’s disease and Parkinson’s disease. Genetics affects the risk of these diseases, but there is a strong non-genetic contribution to the risk, which is poorly understood [1, 2]. There is accumulating evidence (reviewed below) that one of these non-genetic triggers for neurodegeneration is endotoxin. Endotoxin is present in all of us, but levels in blood are very variable and correlate with neurodegeneration. Injection of endotoxin into animals can induce neurodegeneration. So, the hypothesis that endotoxin causes or contributes to neurodegeneration is described and reviewed here, in the hope that a more explicit statement of the hypothesis will encourage testing of it.

## Endotoxin structure and function

Endotoxin is a type of lipopolysaccharide (LPS), consisting of lipid A (usually 6 acyl chains attached to a phosphorylated disaccharide), attached to the ‘core’ (a short sugar chain with various modifications), which is attached to the O-antigen (a long linear chain of sugars of variable length). Endotoxin is a major component of the outer membrane of gram-negative bacteria, with lipid A in the membrane and the O-antigen constituting the outer-facing surface of the bacterium. Soluble endotoxin is released when bacteria are destroyed, but is also released physiologically as outer membrane vesicles.

Different species of gram-negative bacteria have different endotoxin structures, mainly due to differences in (i) the O-antigen, which determines the antigenicity of endotoxin, or (ii) lipid A, which is detected by the main LPS receptor MD2/TLR4 (a complex of myeloid differentiation factor 2 and toll-like receptor 4) and therefore determines inflammation and toxicity [3]. Thus, not all endotoxins are equivalent. LPS toxicity varies depending on lipid A composition, and this depends on bacterial species, strain and environmental conditions [3–5]. For example, the opportunistic lung pathogen *Pseudomonas aeruginosa* changes its lipid A structure from 5 to 6 acyl chains in response to cystic fibrosis, and the resulting hexa-acylated LPS activates MD2/TLR4 much more strongly than penta-acylated LPS [4]. Both *Escherichia coli* and *Bacteroides dorei* are common in human gut, but LPS from *E. coli* has 6 acyl chains in the lipid A, whereas *B. dorei* LPS has 4 or 5 acyl chains, and as a consequence, *E. coli* LPS induces a strong inflammatory response via MD2/TLR4, whereas *B. dorei* LPS does not [5]. Moreover, because *B. dorei* LPS binds but does not activate the MD2/TLR4 receptor complex, it can inhibit the inflammatory response to *E. coli* LPS [5]. Thus, some LPS species are MD2/TLR4 antagonists, and hence anti-inflammatory, as a result of binding but not activating MD2 and/or TLR4 [5].

Gram-negative bacteria, containing endotoxin, are found at very high levels in the mammalian gut (mainly lower intestine) [6]. They are also found in saliva, dental plaque, skin, lungs, respiratory tract and urinary tract. There is very roughly 1 g of endotoxin in the human gut [6], whereas 100 ng of endotoxin injected into blood induces inflammatory activation of the body and brain (Table 1). Humans are orders of magnitude more sensitive to endotoxin than other mammals, such as mice [16].

**Table 1:** Plasma endotoxin levels in different conditions and endotoxin levels causing various effects

Condition	Endotoxin level	Ref:
Healthy humans	10 ± 20 pg/ml	[7, 8]
Atherosclerosis	30 pg/ml	[8]
Peridonitis	45 pg/ml	[9]
Amyotrophic lateral sclerosis	45 pg/ml	[10]
Liver cirrhosis	60 pg/ml	[11]
Alzheimer’s disease	60 pg/ml	[10]
HIV infection/AIDS	70 pg/ml	[12]
Sepsis	500 pg/ml	[13]
Monocyte and endothelial activation	10 pg/ml (added to isolated blood)	[14]
Microglial activation, blood cytokines and sickness behaviour	1 ng/kg (~ 15 pg/ml, iv injected)	[15]

Endotoxin causes inflammatory activation mainly via activating TLR4 (with co-receptor MD2) on the cell surface, resulting in NF-κB transcriptional activation of hundreds of inflammatory genes, including pro-inflammatory cytokines such as TNFα, IL-6 and pro-IL-1β [4, 17, 18]. Lipopolysaccharide binding protein (LBP) is a soluble plasma protein that facilitates the transfer of LPS to membrane-bound CD14, which in turn is required to transfer LPS to TLR4 [18, 19]. Intracellular LPS can also directly activate murine caspase-11 (caspase-4 or caspase-5 in humans), which may then cleave and activate caspase-1, which can cleave pro-IL-1β to IL-1β [20]. Active caspase-1 and caspase-11 can also cleave and activate gasdermin D that permeabilises the plasma membrane allowing IL-1β out, but also killing cells by pyroptosis [20]. Other receptors for endotoxin include RAGE [21], TREM2 [22], the macrophage scavenger receptors [23] and the β_2_ integrins (CD11a/CD18, CD11b/CD18 and CD11c/CD18) [24, 25]. These pattern recognition receptors may function to clear LPS and bacteria expressing LPS from blood and tissues [22, 23, 26], but may also promote inflammation and LPS toxicity [21]. For example, CD11b/CD18 (also known as compliment receptor 3, CR3) mediates microglial ROS production, neurotoxicity and phagocytosis of neurons, and CR3 is implicated in neurodegeneration [27, 28].

High doses of endotoxin in blood (endotoxemia) cause a ‘cytokine storm’, septic shock and death, via activating TLR4, RAGE and caspases [16, 18, 20, 21]. Chronic low doses of endotoxin can either promote low-grade inflammation, tolerance or resolution, depending on other factors [17]. Endotoxin also induces inflammation and other effects indirectly via the pro-inflammatory cytokines TNFα, IL-6 and IL-1β induced by endotoxin. Note, however, that cytokine induction by endotoxin depends on the lipid A structure [3–5].

**Fig. 1:**
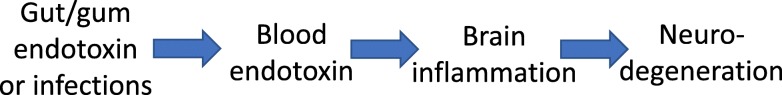
The central pathway of how endotoxin leads to neurodegeneration. Gut endotoxin may enter blood due to leaky gut, e.g. due to alpha-synuclein aggregates. Gum endotoxin may enter blood as a result of gum inflammation or tooth brushing. Blood endotoxin may cause brain inflammation via blood or brain cytokines, or by entering the brain, resulting in neurodegeneration

Endotoxin was originally called ‘endotoxin’, because it was a toxin within the bacteria, to distinguish it from ‘exotoxins’ that were released from bacteria. However, we now know that (i) endotoxin is released by bacteria and (ii) the toxicity of endotoxin is due to the host’s inflammatory over-reaction to it, rather than an intrinsic toxicity to animal cells [3, 16, 18]. LPS constitutes much of the surface of gram-negative bacteria, and thus, animals selected by bacterial diseases have evolved innate immune receptors to detect it with high sensitivity, inducing a strong innate (and adaptive) immune response. This response protects against gram-negative bacterial disease, by promoting the clearance of the bacteria, removing the source of endotoxin. However, (i) if endotoxin levels are too high, they cause acute death by septic shock, and (ii) if endotoxin is not cleared from the blood, it can promote a chronic inflammatory state, which may contribute to multiple chronic diseases [17] (Fig. 1).

## Serum endotoxin

Endotoxin is present in plasma of all healthy humans at very variable levels between 0.01 and 0.5 EU/ml (mean 0.1 ± 0.2 EU/ml), equivalent to about 1 and 50 pg/ml [7, 8]. Note that endotoxin levels are normally measured using a Limulus amebocyte lysate assay (LAL-test) in endotoxin units (EU), a measure of activity not amount, and there are normally between 2 and 50 EU/ng endotoxin, because different endotoxins have different activities (i.e. potencies in the LAL test). Care is required when applying the LAL to blood, as blood components can interfere [29]. Note also that basal plasma levels of endotoxin in rodents are higher than humans (typically 2 EU/ml) and rodents are much less sensitive than humans to injected endotoxin [16].

Serum endotoxin levels are elevated in patients with severe autism [30], liver cirrhosis [11], diabetes [31], cardiovascular disease [8], chronic infection and ageing [32], amyotrophic lateral sclerosis and Alzheimer’s disease [10]. The highest plasma endotoxin levels are found in patients with sepsis, about 500 pg/ml [13].

How much blood endotoxin is required to cause a significant effect in the body and brain? Addition of 10 pg endotoxin/ml to human blood is sufficient to activate monocytes and endothelial cells [14]. Intravenous injection of 1 ng LPS/kg (equivalent to 15 pg/ml distributed through the blood) into healthy human volunteers caused increased blood cytokines (TNFα, IL-6, IL-8, IL-10), increased sickness behaviour (fatigue, headache, muscle pain, shivering) and decreased motivation (alertness, energy, focus, pep, social interest) within 1–3 h, reversing at 4 h [15]. Importantly, this intravenous injection of 1 ng LPS/kg caused a robust microglial activation in most areas of the brain measured by PET (positron emission tomography) imaging of a PBR (peripheral benzodiazepine receptor) ligand 3 h after LPS injection [15]. Thus, a relatively mild dose of blood endotoxin (e.g. less than that found in Alzheimer’s patients) can cause acute microglial activation within the brain. Note, however, that (at least in mice) repeated doses of LPS greatly downregulate body responses to LPS, but brain responses to LPS are less downregulated, partly as a result of epigenetic changes [33]. So, it is difficult to extrapolate the chronic response to blood endotoxin from the acute response. Untreated healthy humans with blood endotoxin at the top end of the normal range have activated monocytes and T cells, with constitutive activation of the transcription factor STAT1 [34]. Thus, the very variable, normal range of blood endotoxin in humans includes levels sufficient to activate the innate immune system.

Where does blood endotoxin come from? Active bacterial infections may produce endotoxin, and for example, urinary tract infections are associated with dementia, delirium and other neuropsychiatric disorders [35]. However, in the absence of infection, endotoxin still crosses the mucosal membranes of gut, gums, nose or lungs, the main source being intestinal permeability [36, 37].

Endotoxin has a hydrophobic lipid A end, so endotoxin aggregates into micelles or vesicles. Within the blood, endotoxin binds to plasma albumin or chylomicrons or high-density lipoproteins (HDL) mediated by LBP, CD14 and APOE [38–40]. Most endotoxin enters the body via the gut and hepatic portal vein, and most of this endotoxin is cleared by the liver [41]. Peripheral blood endotoxin is also cleared and degraded by the liver [39, 41]. So, serious liver disease increases peripheral blood endotoxin to potentially toxic levels (60–80 pg/ml in cirrhosis) [11, 41].

## Peripheral endotoxin can drive brain pathology

Does endotoxin in blood cause neurodegeneration? It is not ethical to test this directly in humans, but this question has been tested in animals.

In rodents, a single intraperitoneal injection of 5 mg LPS/kg causes acute microglial activation in the brains that persists for at least 12 months, and results in loss of dopaminergic neurons in the substantia nigra 10 months later [42, 43]. Multiple doses of 1 mg LPS/kg (over several days), or chronic endotoxin, cause more rapid neurodegeneration and have been used as models of Parkinson’s or Alzheimer’s disease [44, 45]. Direct injection of LPS into the rodent brain is sufficient to induce neuronal loss [44, 46]. Note, however, that (i) these levels of endotoxin would be lethal in humans; (ii) *E. coli* LPS is normally used in these studies, partly because it is the most inflammatory; and (iii) peripheral endotoxin can dramatically increase (‘priming’) or decrease (‘tolerance’) subsequent responses to inflammatory stimuli, including endotoxin, depending on the dose and timing [33, 47].

It is not entirely clear how peripheral endotoxin enters the brain. Endotoxin is found in rat brain in physiological conditions and might cross the blood-brain barrier bound to lipoproteins via lipoprotein transport mechanisms [48]. High-dose endotoxin can induce an increase in blood-brain barrier permeability, allowing plasma components into the brain, potentially resulting in neuroinflammation and neurodegeneration, but also potentially allowing endotoxin into the brain [49–51].

**Fig. 2:**
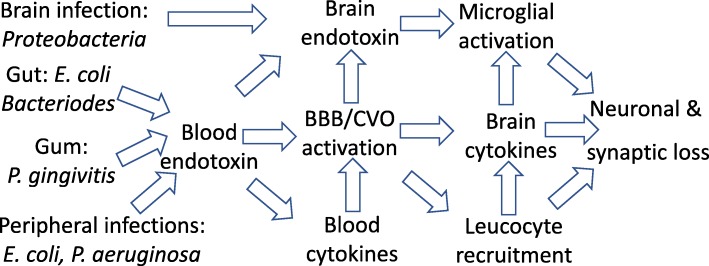
Different species of endotoxin arise from different sources, ending up in the blood or brain. Blood endotoxin increase pro-inflammatory cytokines in blood, and inflammatory activates the blood-brain barrier (BBB) and circumventricular organs (CVO), recruiting leucocytes into the brain and increasing brain cytokines that activate microglia, resulting in synaptic and neuronal loss

Low and medium doses of endotoxin do not change blood-brain barrier permeability and only minimally enter the brain [52], suggesting that peripheral LPS may induce brain inflammation indirectly by (i) LPS activation of peripheral nerves acting centrally; (ii) LPS activation of the blood-brain barrier, which then releases cytokines within the brain, or recruiting immune cells into the brain; or (iii) LPS activation of circumventricular organs. The mechanisms of LPS-induced neurodegeneration and the utility of LPS in modelling neurodegenerative disease are reviewed in [53]. The effects of peripheral endotoxin on the brain can also be mediated by the induced peripheral cytokines, particularly TNFα and IL-1β, which then induce inflammation within the brain [54]. However, sustained brain inflammation in response to blood endotoxin requires brain TLR4, which may be on microglia, endothelium, perivascular macrophages, meninges or circumventricular organs [55]. This implies that the longer-term effect of blood endotoxin on the brain is not mediated by blood cytokines, but may be mediated in part by endotoxin activating the above cells to produce cytokines within the brain (Fig. 2).

Multiple mechanisms have been described by which endotoxin can induce neurodegeneration. Endotoxin stimulates microglia to produce nitric oxide and pro-inflammatory cytokines via activation of TLR4 [56]. If LPS is combined with IFNγ (from recruited T cells), then high levels of iNOS are induced in microglia and astrocytes, and the resulting NO can kill neurons when combined with either hypoxia [57] or superoxide from the NADPH oxidase [58]. However, treatment of glial-neuronal cultures with LPS alone results in little or no neuronal apoptosis or necrosis, but rather progressive loss of neurons over several days due to microglial phagocytosis of stressed-but-viable neurons and blocking the phagocytosis saves the neurons [59]. LPS-activated microglia release NO, superoxide and peroxynitrite that stresses the neurons to reversibly expose phosphatidylserine, which is bound by MFG-E8 released from the astrocytes and microglia, and this MFG-E8 (bound to the stressed neurons) also binds the vitronection receptor, which triggers microglial phagocytosis of these neurons [59, 60]. Engulfment also appears to require UDP released from the stressed neurons to stimulate the P2Y6 receptor on microglia [61]. Thus, blocking the P2Y6 receptor, the vitronection receptor or MFG-E8 prevents LPS-induced neuronal loss in culture or in vivo [59–61]. TNFα can also induce microglia to phagocytose neurons in the absence of LPS [62].

Neuronal loss is often preceded by synaptic loss in neurodegenerative disease, for example in Alzheimer’s disease, and this synaptic loss can be caused by excessive microglial phagocytosis of neurons, driven in part by complement tagging of synapses, triggered by neuroinflammation [28]. Peripheral endotoxin can activate the classical complement system in the brain, resulting in neuronal loss that can be prevented in complement C3-deficient mice [63]. Peripheral endotoxin can also cause microglial activation and loss of brain synapses, and the endotoxin-binding protein APOE2 can protect against this synaptic loss [64]. Synaptic loss may contribute to cognitive deficits in disease, but if excessive might also cause neuronal loss [65].

**Fig. 3:**
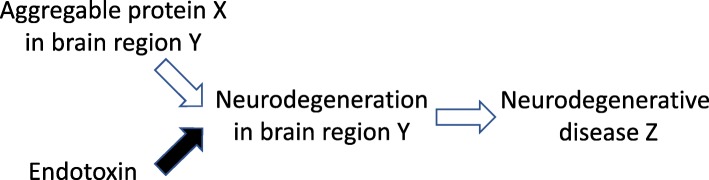
Endotoxin may give rise to different neurodegenerative disease by synergising with different aggregable proteins to induce neurodegeneration. If endotoxin contributes to multiple different diseases, why are these different diseases different? The solution may be a two-hit hypothesis, where the presence of endotoxin or an aggregable protein is not sufficient alone, but together, they induce neurodegeneration and give rise to different neurodegenerative diseases dependent on the particular aggregable protein present and its distribution in the brain. Note that the presence in the brain of an aggregable protein, such as Aβ, Tau or α-synuclein, is not normally sufficient to induce neurodegeneration

High plasma levels of endotoxin can increase permeability of the blood-brain barrier, allowing toxic plasma components, including amyloid β and α-synuclein into the brain [49–51]. Endotoxin may also promote the production or aggregation of amyloid β [66–68], tau [68, 69] and α-synuclein [70, 71]. This suggests the possibility that endotoxin synergises with different aggregable proteins to give different neurodegenerative diseases (Fig. 3).

Blood endotoxin may ‘prime’ microglia to neurodegenerative stimuli (such as amyloid β, tau or α-synuclein), or alternatively, neurodegenerative stimuli (such as aggregating amyloid β, tau or α-synuclein) may prime microglia to endotoxin challenge—either way they synergise to induce neurodegeneration [33, 46]. There is clinical evidence that systemic inflammation triggers neurodegeneration in brains primed by neurodegenerative disease [72]. For example, systemic infections accelerate cognitive decline in Alzheimer’s disease patients [73], and systemic endotoxin precipitates brain pathology in mice expressing prions [74], APP variants [33] or TAU variants [69] or mice with α-synuclein injected in the brain [46].

**Fig. 4:**
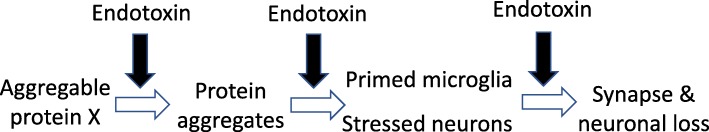
Endotoxin may act at different steps to promote neurodegeneration. (1) Endotoxin may promote aggregates of Aβ, tau, α-synuclein and TDP-43 by inhibiting removal or by increasing production, spread or aggregation (in part by stimulating ROS production). (2) Endotoxin may prime microglia and stress neurons, making them more susceptible to disease-specific agents. (3) Endotoxin may activate microglia, already primed by disease to execute stressed synapses and neurons

Conversely, blood endotoxin may induce tolerance and decrease activation of microglia, which may reduce brain protective functions such as phagocytosis of protein aggregate or debris [17, 33, 75, 76]. The concepts of microglial ‘activation’, ‘priming’ and ‘tolerance’ are loose, but all are reversible states of microglia, mediated by translational and epigenetic changes. In essence, ‘microglial activation’ refers to increased microglial motility, phagocytosis, cytokine release and oxidant production, while ‘microglial priming’ means the microglia are more sensitive to agents causing activation, and ‘microglial tolerance’ means the microglia are less sensitive to agents causing activation [33, 46]. In summary, endotoxin can act at muliple steps to promote neurodegeneration (Fig. 4).

## Endotoxin and Alzheimer’s disease

Alzheimer’s disease (AD) is diagnosed by cognitive and memory deficits during life, and amyloid plaques and tau tangles after death, accompanied by neuroinflammation, synapse loss and neuronal loss. AD is also associated with endotoxin in a number of ways [45, 53, 77]. Mean blood endotoxin levels are increased threefold in AD patients [10]. Brain endotoxin levels are increased two- or threefold in AD patients [78, 79], and endotoxin is also found in AD amyloid plaques [79]. Endotoxin can drive amyloid beta production and aggregation [66–68] and TAU hyperphosphorylation [68, 69]. Eliminating gut bacteria can reduce plaque load and microglial activation in an amyloid model of AD in mice [80].

If AD was in part mediated by endotoxin, then we might expect gene variants associated with AD to interact with endotoxin or endotoxin pathology. The main genetic risk for AD is APOE isoform: APOE2 being protective, APOE3 being neutral and APOE4 being detrimental. Intravenous injection of LPS strongly induces serum ApoE in rodents, and ApoE directly binds LPS, causing LPS to be taken up and degraded by the liver, such that ApoE-deficient mice are more sensitive to LPS toxicity [81, 82]. Humans with the APOE4 variant are more sensitive to injected LPS than those with APOE3, and similarly, mice with endogenous ApoE replaced with APOE4 are more sensitive to LPS than those replaced with APOE3 [83]. Thus, there is a clear and direct link between APOE variants and endotoxin. Sequence variants of the LPS-receptor TLR4 [84] and the LPS-binding receptor TREM2 [22] are also associated with an increased risk of AD, indicating additional genetic links between AD and endotoxin.

People with chronic gum disease (periodontitis) have elevated blood endotoxin [85, 86], a higher risk of Alzheimer’s disease and a faster rate of cognitive decline [87]. The most prevalent bacteria in periodontal diseases are *Porphyromonas gingivalis*. LPS from *P. gingivalis* is less inflammatory than that from *E. coli* on the first encounter; however, on the second encounter, the inflammatory response to *E. coli* is greatly downregulated (‘tolerance’), whereas the response to *P. gingivalis* LPS is not [88]. Chronic oral application of *P. gingivalis* or injection of *P. gingivalis* LPS results in brain inflammation and neurodegeneration in wild-type mice [70, 89]. There is a large increase in bacterial load and bacterial species in AD brains [79, 90], including *P. gingivalis*, which can live intracellularly in glia and neurons [91]. The causes and consequences of these brain bacteria are unclear, but they are a potential source of brain endotoxin, and thus of neuroinflammation and neurodegeneration.

## Endotoxin and Parkinson’s disease

Parkinson’s disease (PD) is diagnosed by motor dysfunctions in life, and after death by α-synuclein aggregates (Lewy bodies) and loss of dopaminergic neurons in the substantia nigra. During PD, α-synuclein aggregation starts in the gut, and one of the earliest symptoms of PD is gut dysfunction [2]. PD patients have increased gastrointestinal permeability [92] and LPS binding protein (LBP) [92], and a proportion of PD patients have elevated blood endotoxin [93]. The gut microbiome of PD patients differs from controls [94]. And the gut microbiome affects motor symptoms in an α-synuclein mouse model of PD, such that eliminating gut bacteria prevents motor deficits, while introducing the gut microbiome from PD patients exacerbates pathology [95]. Gut colonisation with endotoxin-producing *Helicobacter pylori* is associated with PD [96], and eradication of *H. pylori* improved PD symptoms [97, 98].

A single dose of peripheral endotoxin in mice caused increased expression of α-synuclein in neurons of the large intestine, followed by an increase in large intestinal permeability, in a manner similar to that observed in patients with PD [99]. Endotoxin increases α-synuclein production by macrophages [100] and drives α-synuclein fibrillization [70, 71]. A single injection of peripheral endotoxin into wild-type mice and transgenic mice (expressing human A53T mutant α-synuclein) resulted in indistinguishable acute neuroinflammation, but only the transgenic mice treated with endotoxin developed persistent neuroinflammation, aggregated α-synuclein, progressive degeneration of dopamine neurons and Lewy body-like inclusions in nigral neurons [101]. This supports a dual-hit hypothesis for PD: elevated endotoxin plus aggregable α-synuclein results in neurodegeneration. However, endotoxin alone is sufficient to induce loss of dopaminergic neurons in the substantia nigra in mice after a delay of several months [42–44].

## Endotoxin and other brain pathologies

Motor neuron disease is a group of diseases involving neurodegeneration of motor neurons, of which the most common is amyotrophic lateral sclerosis (ALS), which shares genetic and pathological mechanisms with frontotemporal dementia (FTD). In ALS, and many cases of FTD, the motor neurons are filled with abnormal aggregates of TAR DNA-binding protein 43 (TDP-43). Blood endotoxin levels are elevated in ALS patients [10], possibly as a result of gut inflammation and microbiome changes [102]. Addition of LPS to microglia or astrocytes in culture resulted in TDP-43 mislocalization and aggregation, and addition of peripheral LPS to TDP-43(A315T) transgenic mice resulted in TDP-43 aggregation in vivo [103]. This suggests a dual-hit hypothesis for ALS and FTD: increased endotoxin levels plus aggregable TDP-43 results in neurodegeneration.

A variety of roles for endotoxin in multiple sclerosis have been suggested, including (i) promoting microglial damage to myelin and (ii) promoting the presentation of myelin antigens [104].

Plasma endotoxin levels are increased in AIDS, HIV-infected patients and SIV-infected rhesus macaques, as a result of gut-wall damage [12]. HIV infection can progress to HIV-associated dementia, and this correlates with plasma endotoxin levels, which may drive monocyte activation and trafficking into the brain [105].

Chronic alcohol consumption and binge drinking increase serum endotoxin and cytokine levels [106], which might contribute to long-term cognitive deficits.

Bacterial meningitis can be caused by gram-negative bacteria, such as *Neisseria meningitidis*, infecting the brain meninges, often resulting in long-term cognitive problems, and much of the pathology has been attributed to endotoxin [107].

Maternal infection is a risk factor for neurodevelopmental disorders such as autism and schizophrenia. A single pre-natal exposure to LPS (of embryos, for example as a result of maternal infection) or early post-natal exposure can result in long-term activation of brain microglia, lasting into adulthood [108], and behavioural deficits reminiscent of autism or schizophrenia [109]. Serious infections increase the risk of subsequently developing schizophrenia [110], and genetic risk factors for schizophrenia increase microglial phagocytosis of synapses [111], leading to the idea that schizophrenia is triggered by excessive microglial phagocytosis of synapses during adolescence [111], and one potential cause of this is increased endotoxin. Perinatal infections are also associated with autism [109], potentially due to dysfunctional microglial phagocytosis of synapses [112], which could in principle be driven by endotoxin. Blood endotoxin levels are increased in autism [30].

Sepsis is often caused by gram-negative bacteria in the blood, and therefore, blood endotoxin levels can be very high (up to 500 pg/ml [13]). So, in principle, it is a good test of whether high blood endotoxin can cause neurodegeneration in humans. Almost all patients with sepsis have altered consciousness (confusion progressing to delirium and loss of consciousness), and about half of the people surviving serious sepsis have long-term cognitive deficits (called sepsis-associated encephalopathy) [113]. However, it is still unclear that the cognitive deficits are caused by the endotoxin, and it is difficult to extrapolate from a relatively short exposure (days) of high levels of blood endotoxin (as occurs in sepsis) to the long exposure (years) of relatively low blood (or brain) endotoxin that may occur with neurodegenerative disease. Additionally, sepsis is most often caused by bacteria such as *E. coli* or *Pseudomonas aeruginosa* [113], both with highly inflammatory LPS lipid A structures (6 to 7 acyl chains), making it difficult to extrapolate to other diseases.

Liver failure may provide a better test of whether chronically elevated levels of blood endotoxin can cause neurodegeneration in humans. The liver is the main organ for clearing blood endotoxin, so liver failure, as occurs in cirrhosis, elevates blood endotoxin levels to roughly the same level as occurs in Alzheimer’s disease (Table 1). About half of patients with cirrhosis will develop long-term cognitive deficits (called hepatic encephalopathy), progressing from forgetfulness and confusion to coma. However, although blood endotoxin levels correlate with hepatic encephalopathy, so to do blood ammonia and cytokine levels, which are also potentially causal for the encephalopathy, so we still do not know whether endotoxin causes hepatic encephalopathy [114].

## Conclusions

Increased endotoxin is associated with neurodegenerative disease, and increased endotoxin can cause neurodegeneration, but whether neurodegenerative disease is caused by increased endotoxin is not known. Testing this causal link depends on determining whether reducing endotoxin levels or endotoxin actions reduces neurodegenerative disease pathology. Possible means to do this and therefore potential treatment targets include (i) changing the gut microbiome to species with less or less-toxic LPS, (ii) reducing intestinal permeability, (iii) reducing peripheral, peridonatal and/or brain infections, (iv) reducing blood endotoxin levels, (v) reducing LPS actions on, or permeability across, the blood-brain barrier, (vi) inhibiting TLR4 or other LPS receptors or (vii) inhibiting endotoxin-induced microglial activation and neurotoxicity.

The key tests of the plasma endotoxin theory of neurodegeneration are the following: (a) do plasma endotoxin levels correlate with and/or precede neurodegeneration in relevant diseases, and (b) does lowering plasma endotoxin levels in patients reduce subsequent neurodegeneration. We need larger studies of blood endotoxin levels in a variety of neurodegenerative diseases, and we need longitudinal monitoring over the time course of the diseases. Luckily, monitoring blood endotoxin is relatively easy and cheap. However, it will also be important to know the various species of endotoxins in these diseases. Developing treatments that lower blood endotoxin levels in the long term will be difficult, but is likely to be useful for a wide range of conditions, beyond neurodegeneration. LBP, APOE2, polymyxin B or antibodies against LPS could be infused into blood to lower LPS levels, but may be impractical long term. Albumin dialysis is already used to remove endotoxin in patients with liver failure [115] and might be used to test whether lowering endotoxin is beneficial for neurodegeneration, but applying albumin dialysis for several years would be challenging. Vaccines against LPS might be feasible as a longer-term solution.

Animal models will also be important: firstly, to work out how endotoxin causes neurodegeneration; secondly, to test means of blocking endotoxin-induced neurodegeneration; and thirdly, to test potential treatments to lower endotoxin.

## Data Availability

Not applicable.

## Abbreviations

AD: Alzheimer’s disease
AIDS: Acquired immune deficiency syndrome
ALS: Amyotrophic lateral sclerosis
APOE: Apolipoprotein E
APP: Amyloid precursor protein
BBB: Blood-brain barrier
C3: Complement component 3
CVO: Circumventricular organs
EU: Endotoxin units
FTD: Frontotemporal dementia
HDL: High-density lipoproteins
IL: Interleukin
iNOS: Inducible nitric oxide synthase
LAL: Limulus amebocyte lysate
LBP: Lipopolysaccharide binding protein
LPS: Lipopolysaccharide
MD2: Myeloid differentiation factor 2
MFG-E8: Milk fat globulin E8
NO: Nitric oxide
PD: Parkinson’s disease
RAGE: Receptor for advanced glycation endproducts
STAT1: Signal transducer and activator of transcription 1
TDP-43: TAR DNA-binding protein 43
TLR4: Toll-like receptor 4
TNF: Tumour necrosis factor
TREM2: Triggering receptor expressed on myeloid cells
UDP: Uridine diphosphate

## References

[1] VanItallie TB (2017). Alzheimer’s disease: innate immunity gone awry?. Metabolism..

[2] Pfeiffer RF (2018). Gastrointestinal dysfunction in Parkinson’s disease. Curr Treat Options Neurol.

[3] Needham BD, Trent MS (2013). Fortifying the barrier: the impact of lipid A remodelling on bacterial pathogenesis. Nat Rev Microbiol.

[4] Hajjar AM, Ernst RK, Tsai JH, Wilson CB, Miller SI (2002). Human toll-like receptor 4 recognizes host-specific LPS modifications. Nat Immunol.

[5] Vatanen T, Kostic AD, d'Hennezel E, Siljander H, Franzosa EA, Yassour M (2016). Variation in microbiome LPS immunogenicity contributes to autoimmunity in humans. Cell..

[6] Sender R, Fuchs S, Milo R (2016). Revised estimates for the number of human and bacteria cells in the body. PLoS Biol.

[7] Nádházi Z, Takáts A, Offenmüller K, Bertók L (2002). Plasma endotoxin level of healthy donors. Acta Microbiol Immunol Hung.

[8] Wiedermann CJ, Kiechl S, Dunzendorfer S (1999). Association of endotoxemia with carotid atherosclerosis and cardiovascular disease: prospective results from the Bruneck Study. J Am Coll Cardiol.

[9] Kalash D, Vovk A, Huang H, Aukhil I, Wallet SM, Shaddox LM (2015). Influence of periodontal therapy on systemic lipopolysaccharides in children with localized aggressive periodontitis. Pediatr Dent.

[10] Zhang R, Miller RG, Gascon R (2009). Circulating endotoxin and systemic immune activation in sporadic amyotrophic lateral sclerosis (sALS). J Neuroimmunol.

[11] Raparelli V, Basili S, Carnevale R, Napoleone L, Del Ben M, Nocella C, Bartimoccia S, Lucidi C, Talerico G, Riggio O, Violi F (2017). Low-grade endotoxemia and platelet activation in cirrhosis. Hepatology..

[12] Brenchley JM, Price DA, Schacker TW, Asher TE, Silvestri G, Rao S (2006). Microbial translocation is a cause of systemic immune activation in chronic HIV infection. Nat Med.

[13] Opal SM, Scannon PJ, Vincent JL, White M, Carroll SF, Palardy JE, Parejo NA, Pribble JP, Lemke JH (1999). Relationship between plasma levels of lipopolysaccharide (LPS) and LPS-binding protein in patients with severe sepsis and septic shock. J Infect Dis.

[14] Erridge C, Attina T, Spickett CM, Webb DJ (2007). A high-fat meal induces low-grade endotoxemia: evidence of a novel mechanism of postprandial inflammation. Am J Clin Nutr.

[15] Sandiego CM, Gallezot JD, Pittman B, Nabulsi N, Lim K, Lin SF, Matuskey D, Lee JY, O'Connor KC, Huang Y, Carson RE, Hannestad J, Cosgrove KP (2015). Imaging robust microglial activation after lipopolysaccharide administration in humans with PET. Proc Natl Acad Sci U S A.

[16] Fink MP (2014). Animal models of sepsis. Virulence.

[17] Morris MC, Gilliam EA, Li L (2015). Innate immune programing by endotoxin and its pathological consequences. Front Immunol.

[18] Bryant CE, Spring DR, Gangloff M (2010). The molecular basis of the host response to lipopolysaccharide. Nat Rev Microbiol.

[19] Kim SJ, Kim HM (2017). Dynamic lipopolysaccharide transfer cascade to TLR4/MD2 complex via LBP and CD14. BMB Rep.

[20] Pfalzgraff A, Weindl G (2019). Intracellular lipopolysaccharide sensing as a potential therapeutic target for sepsis. Trends Pharmacol Sci.

[21] Yamamoto Y, Harashima A, Saito H, Tsuneyama K, Munesue S, Motoyoshi S (2011). Septic shock is associated with receptor for advanced glycation end products ligation of LPS. J Immunol.

[22] Daws MR, Sullam PM, Niemi EC, Chen TT, Tchao NK, Seaman WE (2003). Pattern recognition by TREM-2: binding of anionic ligands. J Immunol.

[23] Hampton RY, Golenbock DT, Penman M, Krieger M, Raetz CR (1991). Recognition and plasma clearance of endotoxin by scavenger receptors. Nature..

[24] Wright SD, Jong MT (1986). Adhesion-promoting receptors on human macrophages recognize Escherichia coli by binding to lipopolysaccharide. J Exp Med.

[25] Wright SD, Levin SM, Jong MT, Chad Z, Kabbash LG (1989). CR3 (CD11b/CD18) expresses one binding site for Arg-Gly-Asp-containing peptides and a second site for bacterial lipopolysaccharide. J Exp Med.

[26] Fenton MJ, Golenbock DT (1998). LPS-binding proteins and receptors. J Leukoc Biol.

[27] Hou L, Wang K, Zhang C, Sun F, Che Y, Zhao X (2018). Complement receptor 3 mediates NADPH oxidase activation and dopaminergic neurodegeneration through a Src-Erk-dependent pathway. Redox Biol.

[28] Hong S, Beja-Glasser VF, Nfonoyim BM, Frouin A, Li S, Ramakrishnan S (2016). Complement and microglia mediate early synapse loss in Alzheimer mouse models. Science..

[29] Hurley JC (1995). Endotoxemia: methods of detection and clinical correlates. Clin Microbiol Rev.

[30] Emanuele E, Orsi P, Boso M, Broglia D, Brondino N, Barale F, di Nemi SU, Politi P (2010). Low-grade endotoxemia in patients with severe autism. Neurosci Lett.

[31] Jayashree B, Bibin YS, Prabhu D (2014). Increased circulatory levels of lipopolysaccharide (LPS) and zonulin signify novel biomarkers of proinflammation in patients with type 2 diabetes. Mol Cell Biochem.

[32] Glaros TG, Chang S, Gilliam EA, Maitra U, Deng H, Li L (2013). Causes and consequences of low grade endotoxemia and inflammatory diseases. Front Biosci (Schol Ed).

[33] Wendeln AC, Degenhardt K, Kaurani L, Gertig M, Ulas T, Jain G, Wagner J, Häsler LM, Wild K, Skodras A, Blank T, Staszewski O, Datta M, Centeno TP, Capece V, Islam MR, Kerimoglu C, Staufenbiel M, Schultze JL, Beyer M, Prinz M, Jucker M, Fischer A, Neher JJ (2018). Innate immune memory in the brain shapes neurological disease hallmarks. Nature..

[34] Palmer CD, Romero-Tejeda M, Sirignano M (2016). Naturally occurring subclinical endotoxemia in humans alters adaptive and innate immune functions through reduced MAPK and increased STAT1 phosphorylation. J Immunol.

[35] Chae JH, Miller BJ (2015). Beyond urinary tract infections (UTIs) and delirium: a systematic review of UTIs and neuropsychiatric disorders. J Psychiatr Pract.

[36] Bischoff SC, Barbara G, Buurman W (2014). Intestinal permeability – a new target for disease prevention and therapy. BMC Gastroenterol.

[37] Brenchley JM, Douek DC (2012). Microbial translocation across the GI tract. Annu Rev Immunol.

[38] Vreugdenhil ACE, Rousseau CH, Hartung T (2003). Lipopolysaccharide (LPS)-binding protein mediates LPS detoxification by chylomicrons. J Immunol.

[39] Yao Z, Mates JM, Cheplowitz AM (2016). Blood-borne lipopolysaccharide is rapidly eliminated by liver sinusoidal endothelial cells via high-density lipoprotein. J Immunol.

[40] Van Oosten M, Rensen PCN, Van Amersfoort ES (2001). Apolipoprotein E protects against bacterial lipopolysaccharide-induced lethality. J Biol Chem.

[41] Lumsden AB, Henderson JM, Kutner MH (1988). Endotoxin levels measured by a chromogenic assay in portal, hepatic and peripheral venous blood in patients with cirrhosis. Hepatology..

[42] Qin L, Wu X, Block ML, Liu Y, Breese GR, Hong JS, Knapp DJ, Crews FT (2007). Systemic LPS causes chronic neuroinflammation and progressive neurodegeneration. Glia..

[43] Qin L, Liu Y, Hong J-S (2013). NADPH oxidase and aging drive microglial activation, oxidative stress, and dopaminergic neurodegeneration following systemic LPS administration. Glia..

[44] Tufekci KU, Genc S, Genc K (2011). The endotoxin-induced neuroinflammation model of Parkinson’s disease. Parkinsons Dis.

[45] Zakaria R, Wan Yaacob WM, Othman Z, Long I, Ahmad AH, Al-Rahbi B (2017). Lipopolysaccharide-induced memory impairment in rats: a model of Alzheimer’s disease. Physiol Res.

[46] Couch Y, Alvarez-Erviti L, Sibson NR (2011). The acute inflammatory response to intranigral α-synuclein differs significantly from intranigral lipopolysaccharide and is exacerbated by peripheral inflammation. J Neuroinflammation.

[47] Yuan R, Geng S, Li L (2016). Molecular mechanisms that underlie the dynamic adaptation of innate monocyte memory to varying stimulant strength of TLR ligands. Front Immunol.

[48] Vargas-Caraveo A, Sayd A, Maus SR, Caso JR, Madrigal JLM, García-Bueno B, Leza JC (2017). Lipopolysaccharide enters the rat brain by a lipoprotein-mediated transport mechanism in physiological conditions. Sci Rep.

[49] Vutukuri R, Brunkhorst R, Kestner R-I (2018). Alteration of sphingolipid metabolism as a putative mechanism underlying LPS-induced BBB disruption. J Neurochem.

[50] Varatharaj A, Galea I (2017). The blood-brain barrier in systemic inflammation. Brain Behav Immun.

[51] Jaeger LB, Dohgu S, Sultana R (2009). Lipopolysaccharide alters the blood–brain barrier transport of amyloid β protein: a mechanism for inflammation in the progression of Alzheimer’s disease. Brain Behav Immun.

[52] Banks WA, Robinson SM (2010). Minimal penetration of lipopolysaccharide across the murine blood-brain barrier. Brain Behav Immun.

[53] Batista CRA, Gomes GF, Candelario-Jalil E, Fiebich BL, de Oliveira ACP (2019). Lipopolysaccharide-induced neuroinflammation as a bridge to understand neurodegeneration. Int J Mol Sci.

[54] Skelly DT, Hennessy E, Dansereau MA, Cunningham C (2013). A systematic analysis of the peripheral and CNS effects of systemic LPS, IL-1β, TNF-α and IL-6 challenges in C57BL/6 mice. PLoS One.

[55] Chakravarty S, Herkenham M (2005). Toll-like receptor 4 on nonhematopoietic cells sustains CNS inflammation during endotoxemia, independent of systemic cytokines. J Neurosci.

[56] Kinsner A, Boveri M, Hareng L, Brown GC, Coecke S, Hartung T, Bal-Price A (2006). Highly purified lipoteichoic acid induced pro-inflammatory signalling in primary culture of rat microglia through Toll-like receptor 2: selective potentiation of nitric oxide production by muramyl dipeptide. J Neurochem.

[57] Mander P, Borutaite V, Moncada S, Brown GC (2005). Nitric oxide from inflammatory-activated glia synergizes with hypoxia to induce neuronal death. J Neurosci Res.

[58] Mander P, Brown GC (2005). Activation of microglial NADPH oxidase is synergistic with glial iNOS expression in inducing neuronal death: a dual-key mechanism of inflammatory neurodegeneration. J Neuroinflammation.

[59] Neher JJ, Neniskyte U, Zhao JW, Bal-Price A, Tolkovsky AM, Brown GC (2011). Inhibition of microglial phagocytosis is sufficient to prevent inflammatory neuronal death. J Immunol.

[60] Fricker M, Neher JJ, Zhao JW, Théry C, Tolkovsky AM, Brown GC (2012). MFG-E8 mediates primary phagocytosis of viable neurons during neuroinflammation. J Neurosci.

[61] Neher JJ, Neniskyte U, Hornik T, Brown GC (2014). Inhibition of UDP/P2Y6 purinergic signaling prevents phagocytosis of viable neurons by activated microglia in vitro and in vivo. Glia..

[62] Neniskyte U, Vilalta A, Brown GC (2014). Tumour necrosis factor alpha-induced neuronal loss is mediated by microglial phagocytosis. FEBS Lett.

[63] Bodea LG, Wang Y, Linnartz-Gerlach B, Kopatz J, Sinkkonen L, Musgrove R, Kaoma T, Muller A, Vallar L, Di Monte DA, Balling R, Neumann H (2014). Neurodegeneration by activation of the microglial complement-phagosome pathway. J Neurosci.

[64] Zhu Y, Nwabuisi-Heath E, Dumanis SB, Tai LM, Yu C, Rebeck GW, LaDu MJ (2012). APOE genotype alters glial activation and loss of synaptic markers in mice. Glia..

[65] Fricker M, Tolkovsky AM, Borutaite V, Coleman M, Brown GC (2018). Neuronal cell death. Physiol Rev.

[66] Lee JW, Lee YK, Yuk DY, Choi DY, Ban SB, Oh KW, Hong JT (2008). Neuro-inflammation induced by lipopolysaccharide causes cognitive impairment through enhancement of beta-amyloid generation. J Neuroinflammation.

[67] Asti A, Gioglio L (2014). Can a bacterial endotoxin be a key factor in the kinetics of amyloid fibril formation?. J Alzheimers Dis.

[68] Gardner LE, White JD, Eimerbrink MJ (2016). Imatinib methanesulfonate reduces hyperphosphorylation of tau following repeated peripheral exposure to lipopolysaccharide. Neuroscience..

[69] Bhaskar K, Konerth M, Kokiko-Cochran ON (2010). Regulation of tau pathology by the microglial fractalkine receptor. Neuron.

[70] Kim C, Lv G, Lee JS (2016). Exposure to bacterial endotoxin generates a distinct strain of α- synuclein fibril. Sci Rep.

[71] Wang W, Nguyen LTT, Burlak C (2016). Caspase-1 causes truncation and aggregation of the Parkinson’s disease-associated protein α-synuclein. Proc Natl Acad Sci.

[72] Cunningham C (2013). Microglia and neurodegeneration: the role of systemic inflammation. Glia..

[73] Holmes C, Cunningham C, Zotova E, Woolford J, Dean C, Kerr S, Culliford D, Perry VH (2009). Systemic inflammation and disease progression in Alzheimer disease. Neurology..

[74] Cunningham C, Campion S, Lunnon K, Murray CL, Woods JF, Deacon RM, Rawlins JN, Perry VH (2009). Systemic inflammation induces acute behavioral and cognitive changes and accelerates neurodegenerative disease. Biol Psychiatry.

[75] Schaafsma W, Zhang X, van Zomeren KC (2015). Long-lasting pro-inflammatory suppression of microglia by LPS-preconditioning is mediated by RelB-dependent epigenetic silencing. Brain Behav Immun.

[76] Pardon MC (2015). Lipopolysaccharide hyporesponsiveness: protective or damaging response to the brain?. Romanian J Morphol Embryol.

[77] Zhan X, Stamova B, Sharp FR (2018). Lipopolysaccharide associates with amyloid plaques, neurons and oligodendrocytes in Alzheimer’s disease brain: a review. Front Aging Neurosci.

[78] Zhao Y, Jaber V, Lukiw WJ (2017). Secretory products of the human GI tract microbiome and their potential impact on Alzheimer’s disease (AD): detection of lipopolysaccharide (LPS) in AD hippocampus. Front Cell Infect Microbiol.

[79] Zhan X, Stamova B, Jin L-W (2016). Gram-negative bacterial molecules associate with Alzheimer disease pathology. Neurology..

[80] Minter MR, Zhang C, Leone V, Ringus DL, Zhang X, Oyler-Castrillo P (2016). Antibiotic-induced perturbations in gut microbial diversity influences neuro-inflammation and amyloidosis in a murine model of Alzheimer’s disease. Sci Rep.

[81] Rensen PC, Oosten M, Bilt E, Eck M, Kuiper J, Berkel TJ (1997). Human recombinant apolipoprotein E redirects lipopolysaccharide from Kupffer cells to liver parenchymal cells in rats In vivo. J Clin Invest.

[82] Van Oosten M, Rensen PC, Van Amersfoort ES, Van Eck M, Van Dam AM, Breve JJ, Vogel T, Panet A, Van Berkel TJ, Kuiper J (2001). Apolipoprotein E protects against bacterial lipopolysaccharide-induced lethality. A new therapeutic approach to treat gram-negative sepsis. J Biol Chem.

[83] Gale SC, Gao L, Mikacenic C, Coyle SM, Rafaels N, Murray Dudenkov T, Madenspacher JH, Draper DW, Ge W, Aloor JJ, Azzam KM, Lai L, Blackshear PJ, Calvano SE, Barnes KC, Lowry SF, Corbett S, Wurfel MM, Fessler MB (2014). APOε4 is associated with enhanced in vivo innate immune responses in human subjects. J Allergy Clin Immunol.

[84] Chen YC, Yip PK, Huang YL, Sun Y, Wen LL, Chu YM, Chen TF (2012). Sequence variants of toll like receptor 4 and late-onset Alzheimer’s disease. PLoS One.

[85] Wahaidi VY, Kowolik MJ, Eckert GJ, Galli DM (2011). Endotoxemia and the host systemic response during experimental gingivitis. J Clin Periodontol.

[86] Shaddox LM, Wiedey J, Calderon NL, Magnusson I, Bimstein E, Bidwell JA, Zapert EF, Aukhil I, Wallet SM (2011). Local inflammatory markers and systemic endotoxin in aggressive periodontitis. J Dent Res.

[87] Ide M, Harris M, Stevens A, Sussams R, Hopkins V, Culliford D, Fuller J, Ibbett P, Raybould R, Thomas R, Puenter U, Teeling J, Perry VH, Holmes C (2016). Periodontitis and cognitive decline in Alzheimer’s disease. PLoS One.

[88] Martin M, Katz J, Vogel SN, Michalek SM (2001). Differential induction of endotoxin tolerance by lipopolysaccharides derived from Porphyromonas gingivalis and Escherichia coli. J Immunol.

[89] Zhang J, Yu C, Zhang X, Chen H, Dong J, Lu W, Song Z, Zhou W (2018). Porphyromonas gingivalis lipopolysaccharide induces cognitive dysfunction, mediated by neuronal inflammation via activation of the TLR4 signaling pathway in C57BL/6 mice. J Neuroinflammation.

[90] Emery DC, Shoemark DK, Batstone TE, Waterfall CM, Coghill JA, Cerajewska TL, Davies M, West NX, Allen SJ (2017). 16S rRNA next generation sequencing analysis shows bacteria in Alzheimer’s post-mortem brain. Front Aging Neurosci.

[91] Ilievski V, Zuchowska PK, Green SJ, Toth PT, Ragozzino ME, Le K, Aljewari HW, O'Brien-Simpson NM, Reynolds EC, Watanabe K (2018). Chronic oral application of a periodontal pathogen results in brain inflammation, neurodegeneration and amyloid beta production in wild type mice. PLoS One.

[92] Forsyth CB, Shannon KM, Kordower JH (2011). Increased intestinal permeability correlates with sigmoid mucosa alpha-synuclein staining and endotoxin exposure markers in early Parkinson’s disease. PLoS One.

[93] Wijeyekoon RS. The biological basis of heterogeneity in Parkinson’s disease - insights from an innate immune perspective. Doctoral thesis, University of Cambridge, 2018, doi: 10.17863/CAM.30569

[94] Scheperjans F, Aho V, Pereira PAB (2015). Gut microbiota are related to Parkinson’s disease and clinical phenotype. Mov Disord.

[95] Sampson TR, Debelius JW, Thron T (2016). Gut microbiota regulate motor deficits and neuroinflammation in a model of Parkinson’s disease. Cell..

[96] Shen Xiaoli, Yang Huazhen, Wu Yili, Zhang Dongfeng, Jiang Hong (2017). Meta-analysis: Association of Helicobacter pylori infection with Parkinson's diseases. Helicobacter.

[97] Rees K, Stowe R, Patel S, Ives N, Breen K, Clarke CE, Ben‐Shlomo Y. Helicobacter pylori eradication for Parkinson's disease. Cochrane Database Syst Rev. 2011;11:CD008453. 10.1002/14651858.CD008453.pub2 PMC13126632 22071847

[98] Liu H, Su W, Li S (2017). Eradication of Helicobacter pylori infection might improve clinical status of patients with Parkinson’s disease, especially on bradykinesia. Clin Neurol Neurosurg.

[99] Kelly LP, Carvey PM, Keshavarzian A, Shannon KM, Shaikh M, Bakay RA, Kordower JH (2014). Progression of intestinal permeability changes and alpha-synuclein expression in a mouse model of Parkinson’s disease. Mov Disord.

[100] Tanji K, Mori F, Imaizumi T (2002). Upregulation of alpha-synuclein by lipopolysaccharide and interleukin-1 in human macrophages. Pathol Int.

[101] Gao HM, Zhang F, Zhou H, Kam W, Wilson B, Hong JS (2011). Neuroinflammation and α-synuclein dysfunction potentiate each other, driving chronic progression of neurodegeneration in a mouse model of Parkinson’s disease. Environ Health Perspect.

[102] Rowin J, Xia Y, Jung B, Sun J (2017). Gut inflammation and dysbiosis in human motor neuron disease. Physiol Rep.

[103] Correia AS, Patel P, Dutta K, Julien JP (2015). Inflammation induces TDP-43 mislocalization and aggregation. PLoS One.

[104] Hänninen A (2017). Infections in MS: an innate immunity perspective. Acta Neurol Scand.

[105] Ancuta P, Kamat A, Kunstman KJ, Kim EY, Autissier P, Wurcel A, Zaman T, Stone D, Mefford M, Morgello S, Singer EJ, Wolinsky SM, Gabuzda D (2008). Microbial translocation is associated with increased monocyte activation and dementia in AIDS patients. PLoS One.

[106] Bala S, Marcos M, Gattu A, Catalano D, Szabo G (2014). Acute binge drinking increases serum endotoxin and bacterial DNA levels in healthy individuals. PLoS One.

[107] Brandtzaeg P, van Deuren M (2012). Classification and pathogenesis of meningococcal infections. Methods Mol Biol.

[108] O'Loughlin E, Pakan JMP, Yilmazer-Hanke D, McDermott KW (2017). Acute in utero exposure to lipopolysaccharide induces inflammation in the pre- and postnatal brain and alters the glial cytoarchitecture in the developing amygdala. J Neuroinflammation.

[109] Custódio CS, Mello BSF, Filho AJMC, de Carvalho Lima CN, Cordeiro RC, Miyajima F, Réus GZ, Vasconcelos SMM, Barichello T, Quevedo J, de Oliveira AC, de Lucena DF, Macedo DS (2018). Neonatal immune challenge with lipopolysaccharide triggers long-lasting sex- and age-related behavioral and immune/neurotrophic alterations in mice: relevance to autism spectrum disorders. Mol Neurobiol.

[110] Benros ME, Nielsen PR, Nordentoft M, Eaton WW, Dalton SO, Mortensen PB (2011). Autoimmune diseases and severe infections as risk factors for schizophrenia: a 30-year population-based register study. Am J Psychiatry.

[111] Sekar A, Bialas AR, de Rivera H, Davis A, Hammond TR, Kamitaki N, Tooley K, Presumey J, Baum M, Van Doren V, Genovese G, Rose SA, Handsaker RE, Daly MJ, Carroll MC, Stevens B, McCarroll SA, Schizophrenia Working Group of the Psychiatric Genomics Consortium (2016). Schizophrenia risk from complex variation of complement component 4. Nature..

[112] Zhan Y, Paolicelli RC, Sforazzini F, Weinhard L, Bolasco G, Pagani F, Vyssotski AL, Bifone A, Gozzi A, Ragozzino D, Gross CT (2014). Deficient neuron-microglia signaling results in impaired functional brain connectivity and social behavior. Nat Neurosci.

[113] Widmann CN, Heneka MT (2014). Long-term cerebral consequences of sepsis. Lancet Neurol.

[114] Jain L, Sharma BC, Sharma P, Srivastava S, Agrawal A, Sarin SK (2012). Serum endotoxin and inflammatory mediators in patients with cirrhosis and hepatic encephalopathy. Dig Liver Dis.

[115] García Martínez JJ, Bendjelid K (2018). Artificial liver support systems: what is new over the last decade?. Ann Intensive Care.

